# β-arrestin2 deficiency protects against hepatic fibrosis in mice and prevents synthesis of extracellular matrix

**DOI:** 10.1038/s41419-020-2596-8

**Published:** 2020-05-21

**Authors:** Wu-Yi Sun, Yuan-Jing Gu, Xin-Ran Li, Jia-Chang Sun, Jia-Jia Du, Jing-Yu Chen, Yang Ma, Qing-Tong Wang, Wei Wei

**Affiliations:** Institute of Clinical Pharmacology of Anhui Medical University, Key Laboratory of Anti-inflammatory and Immune Medicine, Ministry of Education, Anhui Collaborative Innovation Center of Anti-Inflammatory and Immune Medicine, 230032 Hefei, Anhui Province China

**Keywords:** Gastrointestinal diseases, Medical research

## Abstract

Hepatic fibrosis is a disease of the wound-healing response following chronic liver injury, and activated hepatic stellate cells (HSCs) play a crucial role in the progression of hepatic fibrosis. β-arrestin2 functions as a multiprotein scaffold to coordinate complex signal transduction networks. Although β-arrestin2 transduces diverse signals in cells, little is known about its involvement in the regulation of liver fibrosis. Our current study utilized a porcine serum-induced liver fibrosis model and found increased expression of β-arrestin2 in hepatic tissues with the progression of hepatic fibrosis, which was positively correlated with collagen levels. Furthermore, changes in human fibrotic samples were also observed. We next used β-arrestin2^−/−^ mice to demonstrate that β-arrestin2 deficiency ameliorates CCl_4_-induced liver fibrosis and decreases collagen deposition. The in vitro depletion and overexpression experiments showed that decreased β-arrestin2 inhibited HSCs collagen production and elevated TβRIII expression, thus downregulating the TGF-β1 pathway components Smad2, Smad3 and Akt. These findings suggest that β-arrestin2 deficiency ameliorates liver fibrosis in mice, and β-arrestin2 may be a potential treatment target in hepatic fibrosis.

## Introduction

Hepatic fibrosis is a common final pathway of a variety of chronic liver diseases and is often associated with severe morbidity and mortality. The pathogenesis of hepatic fibrosis is characterized by the excessive accumulation of extracellular matrix (ECM) and formation of fibrous scars, which lead to destruction of the normal liver parenchyma^1^. The activation of hepatic stellate cells (HSCs) is reported to play a crucial role in the formation of liver fibrosis and is a major cellular source of matrix proteins^2^. At the cellular level, transforming growth factor-β1 (TGF-β1) is critical in the progression of liver fibrosis due to its role in regulating ECM synthesis, HSC proliferation, and apoptosis. Following liver injury, HSCs are activated and secrete latent TGF-β, which forms an autocrine positive feedback loop to induce fibrogenesis through Smad2/3^3^. TGF-β1 functions by binding to three receptors: type I (TβRI), type II (TβRII) and type III (TβRIII) TGF-β1 receptor. Both Smad-dependent pathways (such as Smad2 and Smad3) and several Smad-independent pathways, such as mitogen-activated protein kinases (MAPKs) and phosphatidylinositol 3-kinase (PI3K)/Akt, are critical for TGF-β1-mediated signalling^4^. Currently, clinical reports suggest that advanced liver fibrosis is potentially reversible. Therefore, it is crucial to develop effective antifibrotic strategies.

There are four members of the arrestin family: β-arrestin1, β-arrestin2, x-arrestin, and s-arrestin. β-arrestin1 and β-arrestin2 are extensively expressed; however, x-arrestin and s-arrestin are mainly expressed in the visual system. β-arrestin2 transduces G protein-coupled receptor (GPCR) signals and mediates desensitization, internalization, degradation and recycling of GPCR^5^. Mounting evidence suggests that, in addition to regulating GPCR signals, β-arrestin2 also regulates the signalling and/or endocytosis of non-GPCR, including extracellular regulated kinase (ERK), JNK, TβRIII, and interleukin-1 receptor^6^. Aberrant expression of β-arrestins has been reported in some fibrotic diseases, including cardiac fibrosis^7^, pulmonary fibrosis^8^ and renal fibrosis^9^.

Although β-arrestin2 transduces multiple signals in cells, its role in the modulation of liver fibrosis is unclear. We previously reported that β-arrestin2 depletion diminishes HSC mitogenic signalling and proliferation in vitro^10^. However, the potential of β-arrestin2 in the development of liver fibrosis in vivo and ECM synthesis has not been investigated. To that end, the present study utilized a porcine serum (PS)-induced liver fibrosis model and found increased expression of β-arrestin2 in hepatic tissues with the progression of hepatic fibrosis, which was positively correlated with collagen levels. Furthermore, changes in human fibrotic samples were also observed. We next used β-arrestin2^−/−^ mice to further demonstrate that β-arrestin2 deficiency ameliorates carbon tetrachloride (CCl_4_)-induced liver fibrosis and decreases ECM deposition. In vitro depletion and overexpression experiments showed that decreased β-arrestin2 inhibited collagen production by HSCs and elevated TβRIII expression, thus downregulating the TGF-β1 pathway components Smad2, Smad3 and Akt. Taken together, these findings suggest that β-arrestin2 is a potential treatment target in hepatic fibrosis.

## Results

### β-arrestin2 expression correlated with collagen production during fibrosis development

**Fig. 1:**
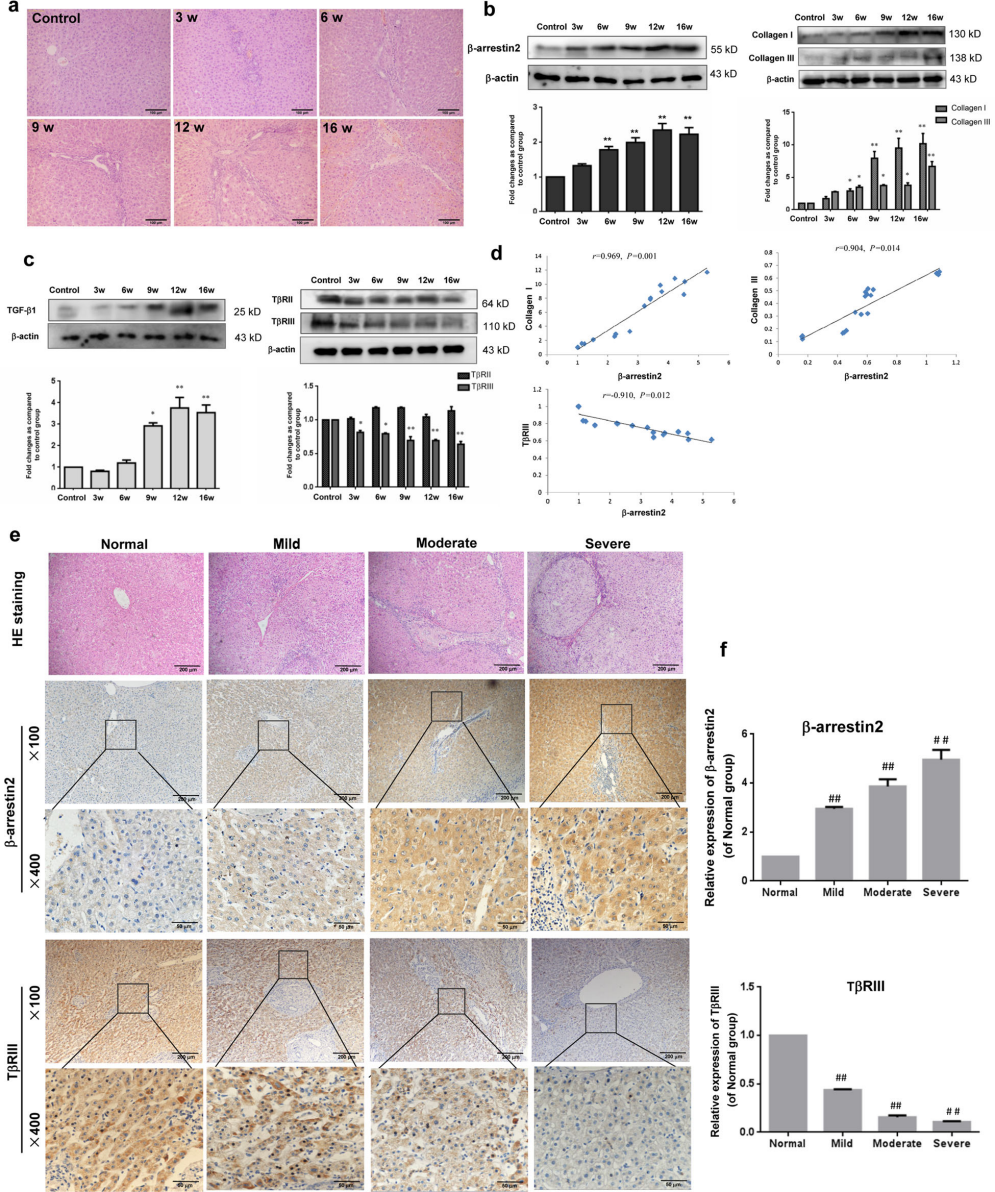
β-arrestin2 expression is associated with collagen production in liver fibrosis development. **a** Representative photographs of HE staining from control rats and 3, 6, 9, 12, 16 weeks after PS injection (*n* = 8 in each group, scale bar = 100 μm). **b** Time course analysis of β-arrestin2 and collagen I, collagen III expression by Western blot in PS-induced liver fibrosis rats. **c** TGF-β1, TβRII, TβRIII protein expressions in liver tissues of fibrotic rats at different time points, and the protein levels were normalized to β-actin for the Western blots from the same lysate. Densitometry values in the histograms were expressed as -fold change relative to the control, which was assigned a value of 1. The data from at least four independent experiments are shown as mean ± SD. *P < 0.05, **P < 0.01 vs. control group. **d** The correlation analysis of β-arrestin2 and collagen I, collagen III, TβRIII expression in liver fibrosis. **e** Immunohistochemical analysis of β-arrestin2 and TβRIII expression in human samples from normal liver and different histological grades of liver fibrosis (scale bar = 200 μm for ×100 and 50 μm for ×400). Top panel is HE staining for a serial section (scale bar = 200 μm). **f** Bar graph of the relative positive optical density values of β-arrestin2 and TβRIII in the hepatic tissues (*n* = 8 in normal group, *n* = 10 in mild group, and *n* = 9 in moderate group, severe group). ^*##*^*P* < 0.01 vs. normal group.

To study the dynamic expression of β-arrestin2 in vivo, we established a PS-induced liver fibrosis model to investigate the changes during fibrosis progression. Hematoxylin-eosin (HE) staining showed that at 6 weeks after PS injection, the liver displayed disordered hepatic cords, massive infiltration of inflammatory cells and considerable collagen deposition. Between 9 and 16 weeks, the apparent fibrous septa showed radial extension, resulting in the formation of pseudolobules (Fig. 1a). Western blot analysis showed that β-arrestin2 protein levels in the liver tissues of fibrotic rats increased with the progression of fibrosis. Furthermore, the deposition of collagen I and collagen III in the rat liver correspondingly increased (Fig. 1b).

Considering the close association of TGF-β1 with collagen production and hepatic fibrosis, we investigated the expression of TGF-β1 and its receptors TβRII and TβRIII in liver tissues. Western blot analysis showed that TGF-β1 expression was upregulated compared with that in the normal control group with increasing severity of hepatic fibrosis. However, the expression of TβRIII in PS-injected rats was significantly lower than that in the normal control group. We found that TβRII expression was not significantly changed with the progression of fibrosis (Fig. 1c). Correlation analysis revealed that β-arrestin2 expression in fibrotic livers was positively associated with collagen I and collagen III levels but negatively associated with TβRIII expression (Fig. 1d). Collectively, these data revealed the correlation between β-arrestin2 expression and collagen production in fibrosis development.

### β-arrestin2 was frequently upregulated in patients with liver fibrotic diseases

To further verify the role of β-arrestin2 in liver fibrosis, we examined the β-arrestin2 expression profiles in human samples by immunohistochemistry. β-arrestin2 staining intensity showed a clearly increasing trend in mild fibrosis and showed significantly enhanced levels in severely fibrotic patients compared with that of samples from control livers. However, TβRIII was significantly downregulated in fibrotic livers (Fig. 1e, f).

### β-arrestin2 deficiency ameliorated liver fibrosis in mice

**Fig. 2:**
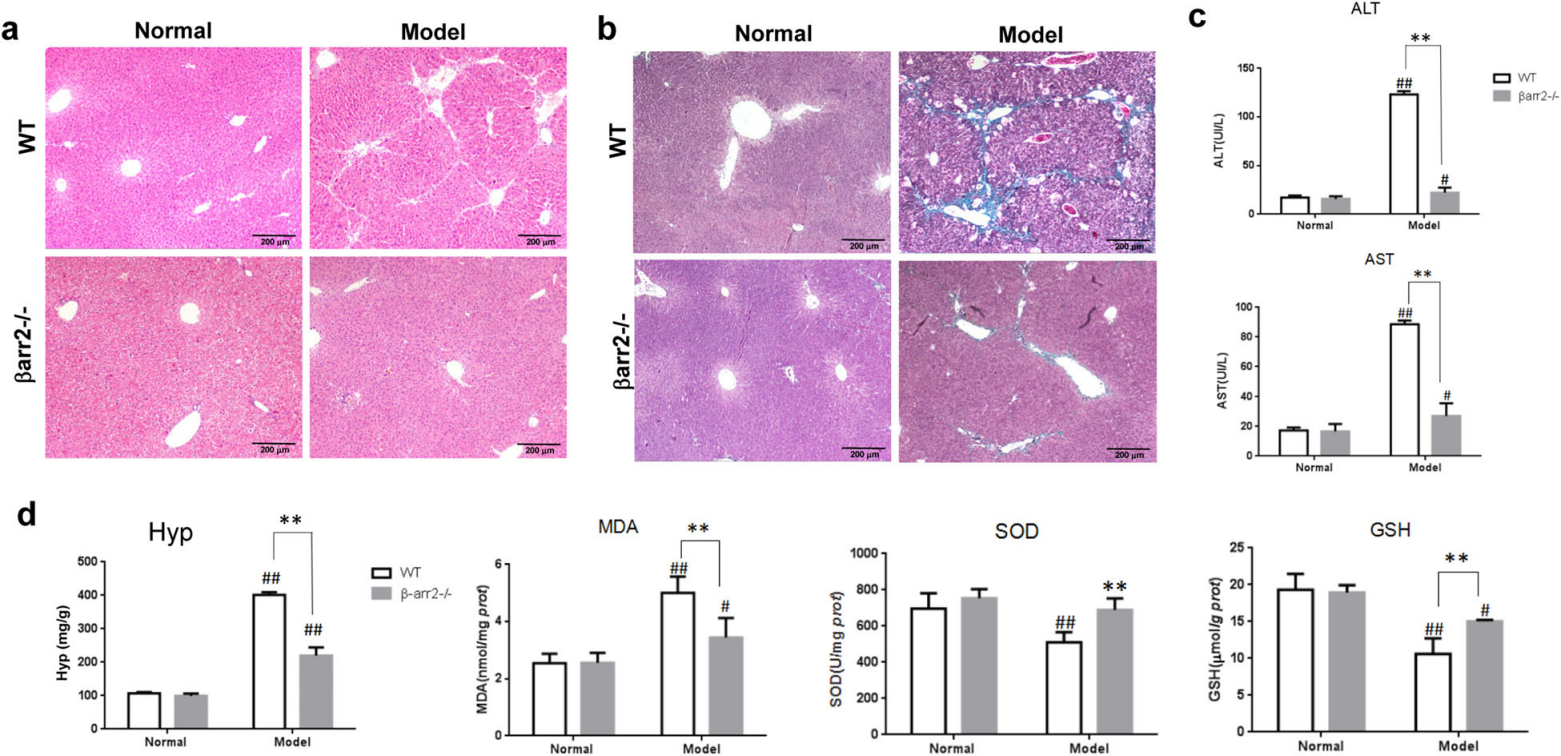
β-arrestin2 deficiency ameliorated CCl_4_-induced liver fibrosis in mice. Liver sections from the WT and β-arrestin2^−/−^ mice at 6 weeks post CCl_4_ injection were stained with HE (**a**) or Masson’s trichrome (**b**). Scale bar = 200 μm. **c** Serum ALT and AST activities of β-arrestin2^−/−^ and WT mice 6 weeks after intraperitoneal injection of CCl_4_ 5 mL/kg (*n* = 8 in each group). **d** Effect of β-arrestin2 deficiency on the hydroxyproline content, lipid peroxidation products MDA, and SOD, GSH levels of liver homogenates in CCl_4_-induced liver fibrotic mice (*n* = 8 in each group). ^#^*P* < 0.05, ^##^*P* < 0.01 vs. normal group; **P < 0.01 vs. WT model group.

To determine the contribution of β-arrestin2 to hepatic fibrosis, we used β-arrestin2^−/−^ mice in the CCl_4_ mouse model of liver fibrosis. Injection of CCl_4_ resulted in serious hepatic steatosis, necrosis, severe architectural changes and excessive collagen accumulation in WT mice. However, the livers of β-arrestin2^−/−^ mice showed minimal collagen accumulation (Fig. 2a, b). Consistent with the liver histology results, β-arrestin2^−/−^ mice showed obviously reduced hydroxyproline in liver homogenates compared with that of WT mice upon CCl_4_ treatment (Fig. 2d).

Increased levels of alanine aminotransferase (ALT) and aspartate aminotransferase (AST) are conventional indicators of liver injury. An obvious increase in the activity of these two enzymes was detected in WT model mice, while a significant reduction was observed in β-arrestin2^−/−^ mice (Fig. 2c). Since lipid peroxidation is considered an important factor in CCl_4_-induced hepatotoxicity, we examined malondialdehyde (MDA), superoxide dismutase (SOD) and glutathione (GSH) levels in liver homogenates. WT mice had a significant increase in MDA levels and a decrease in SOD and GSH levels. MDA levels were significantly decreased and SOD, GSH levels were increased in the livers of β-arrestin2^−/−^ mice compared with those of WT mice (Fig. 2d). These data indicate that β-arrestin2-deficient mice are protected from redundant collagen deposition and architectural changes in the liver that commonly occur upon CCl_4_ treatment.

### β-arrestin2 deficiency inhibited the activation of T cells

**Fig. 3:**
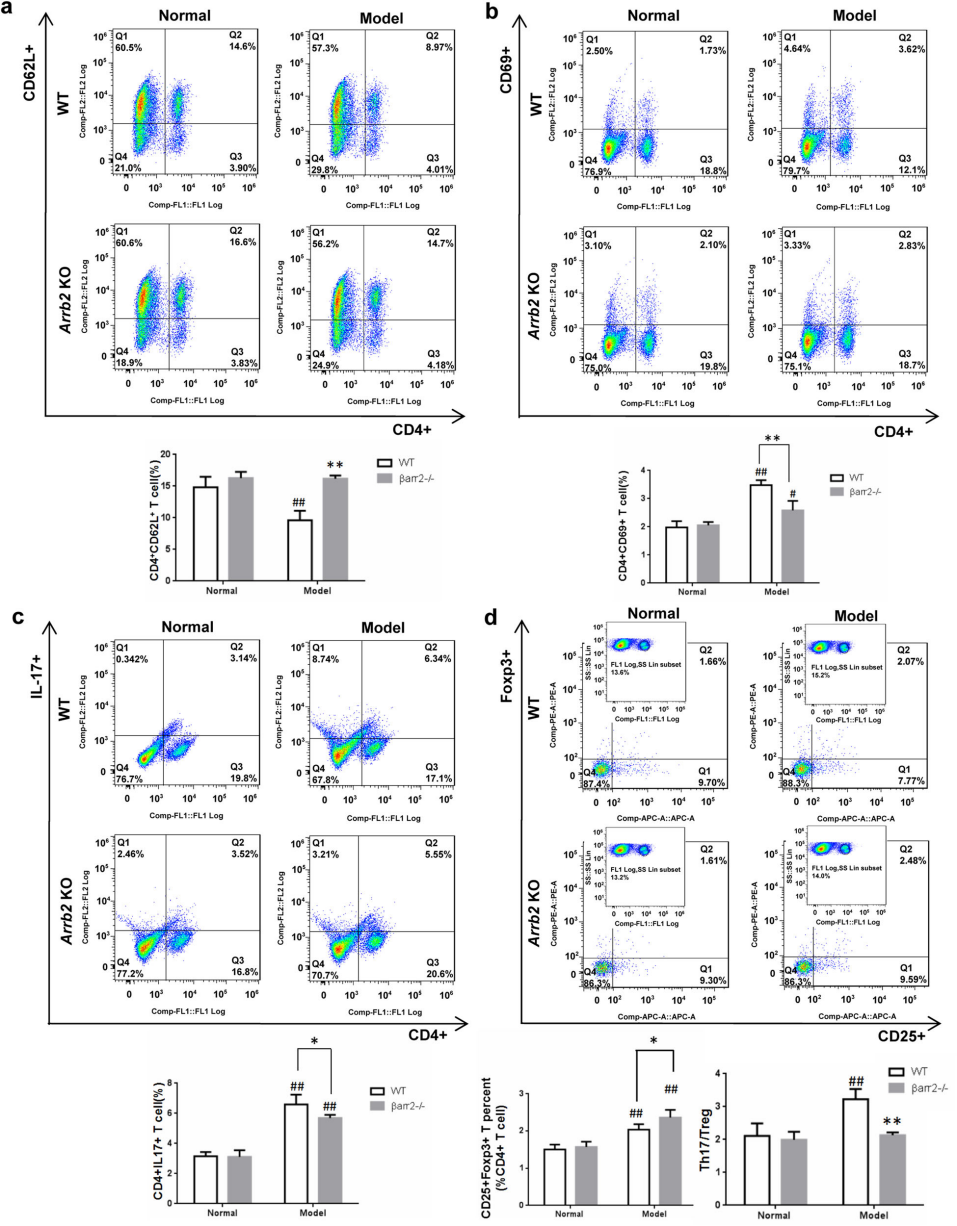
β-arrestin2 deficiency inhibited activation of T cells. Molecule expressions on T cell surface were detected using a flow cytometer. The representative flow cytometry dot plot and bar graph of CD4^+^CD62L^+^ naïve T cells (**a**), CD4^+^CD69^+^ activated T cells (**b**), CD4^+^IL-17^+^ Th17 cells (**c**), CD4^+^CD25^+^Foxp3^+^ Treg (**d**). ^#^*P* < 0.05, ^##^*P* < 0.01 vs. normal group; *P < 0.05, **P < 0.01 vs. WT model group.

Activated T cells also play a crucial role in the pathogenesis of hepatic fibrosis^11^. To determine if the loss of β-arrestin2 alters T cell subsets, the subsets of naïve T cells (CD4^+^CD62L^+^), activated T cells (CD4^+^CD69^+^), Th17 cells (CD4^+^IL-17^+^) and regulatory T (Treg) cells (CD4^+^CD25^+^Foxp3^+^) were examined. The results showed that in the spleens of WT model mice, activated T cells and the Th17/Treg ratio significantly increased, while naïve T cells decreased. β-arrestin2 deficiency led a decrease in activated T cell populations and Th17/Treg ratio and increased naïve T cells in the spleens of fibrotic mice (Fig. 3a–d). These data suggest that the inflammatory response after CCl_4_ treatment is attenuated in β-arrestin2^−/−^ mice.

### β-arrestin2 deficiency reduced collagen production and TGF-β1 signalling in fibrotic mice

**Fig. 4:**
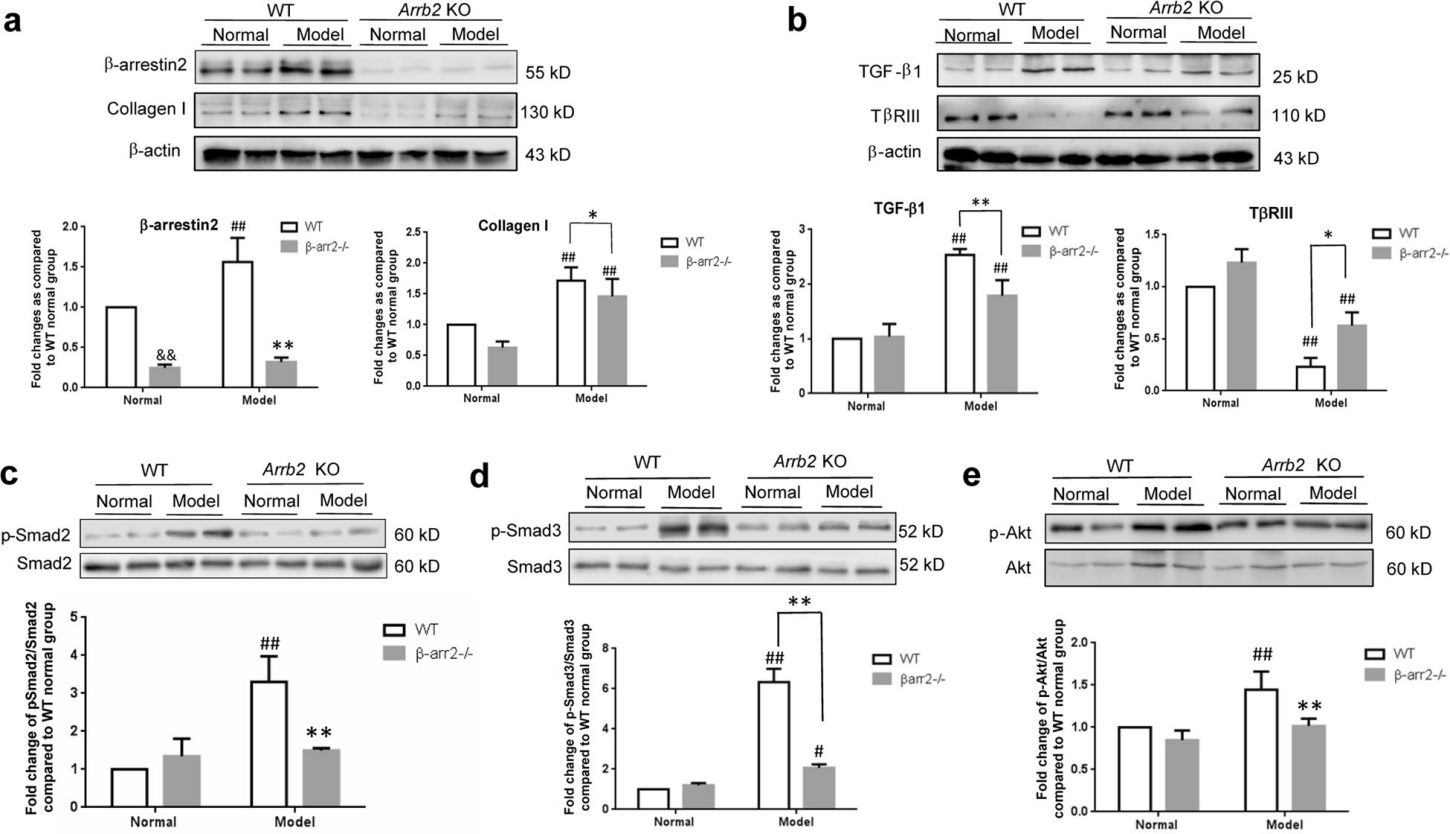
β-arrestin2 deficiency reduced collagen production and TGF-β1 signalling in fibrotic mice. Western blot analysis of β-arrestin2 and collagen I (**a**), TGF-β1 and its co-receptor TβRIII (**b**), p-Smad2 (**c**), p-Smad3 (**d**), p-Akt (**e**) from liver tissue protein extracts of WT and β-arrestin2^-/-^ mice (*n* = 8 per group). Densitometry values in the histograms were expressed as -fold change relative to WT normal group, which was assigned a value of 1. ^&&^*P* < 0.01 vs. WT normal group; ^#^*P* < 0.05, ^##^*P* < 0.01 vs. normal group; *P < 0.05, **P < 0.01 vs. WT model group.

To ascertain whether the alleviation of fibrosis in β-arrestin2^−/−^ mice is due to the loss of TGF-β1 responsiveness, the expression of TGF-β1 downstream signalling molecules was detected. Importantly, the β-arrestin2 level was also elevated in the WT CCl_4_-induced fibrosis model (Fig. 4a). These observations were consistent with a model of PS-induced fibrosis as previously mentioned. As shown in Fig. 4a–e, the expression of collagen I, TGF-β1, p-Smad2, p-Smad3, and p-Akt was decreased, whereas TβRIII expression was increased in β-arrestin2^−/−^ mice compared with those of WT mice. These data suggest that the prevention of liver fibrosis in the absence of β-arrestin2 may be due to downregulating TGF-β1 signalling.

### Gene silencing of TβRIII enhanced TGF-β1-induced collagen production

**Fig. 5:**
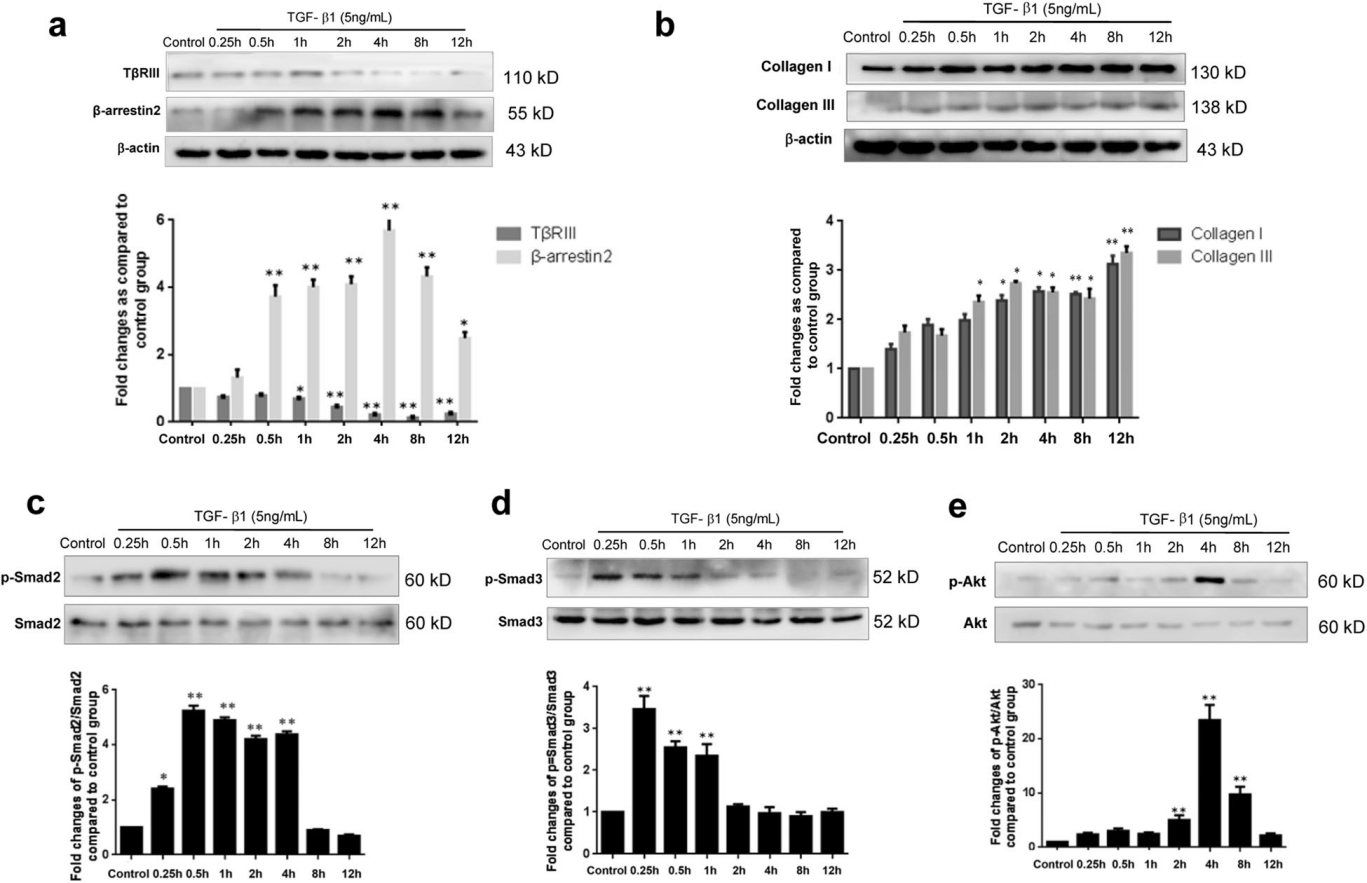
Time-course expression of β-arrestin2, TβRIII and its downstream signalling in HSCs stimulated with TGF-β1. HSCs were incubated with 5 ng/mL TGF-β1 for the time indicated in the figure and was analyzed by Western blot. HSCs without any TGF-β1 stimulation were used as the baseline control. Representative blot and the histogram corresponding to the quantitative analysis of β-arrestin2 and TβRIII (**a**), collagen I and collagen III (**b**), p-Smad2 (**c**), p-Smad3 (**d**), p-Akt (**e**) are shown. Densitometry values in the histograms were expressed as -fold change relative to the control, which was assigned a value of 1. The data are presented as the mean ± SD from at least four independent experiments. *P < 0.05, **P < 0.01 vs. control group.

Considering that TGF-β1 plays a pivotal role in hepatic fibrosis^3^, TGF-β1-stimulated HSCs were used in this in vitro study. Time-course expression of β-arrestin2, TβRIII and its downstream signalling components in HSC-T6 cells that were stimulated with TGF-β1 was detected. As shown in Fig. 5a, β-arrestin2 expression was progressively increased in HSCs after stimulation with TGF-β1 for 0.5 h and peaked 4 h. However, TβRIII expression was decreased under TGF-β1 stimulation. As expected, TGF-β1 treatment induced increases in collagen I and collagen III levels (Fig. 5b), which was accompanied by upregulation of p-Smad2, p-Smad3 and p-Akt (Fig. 5c–e).

**Fig. 6:**
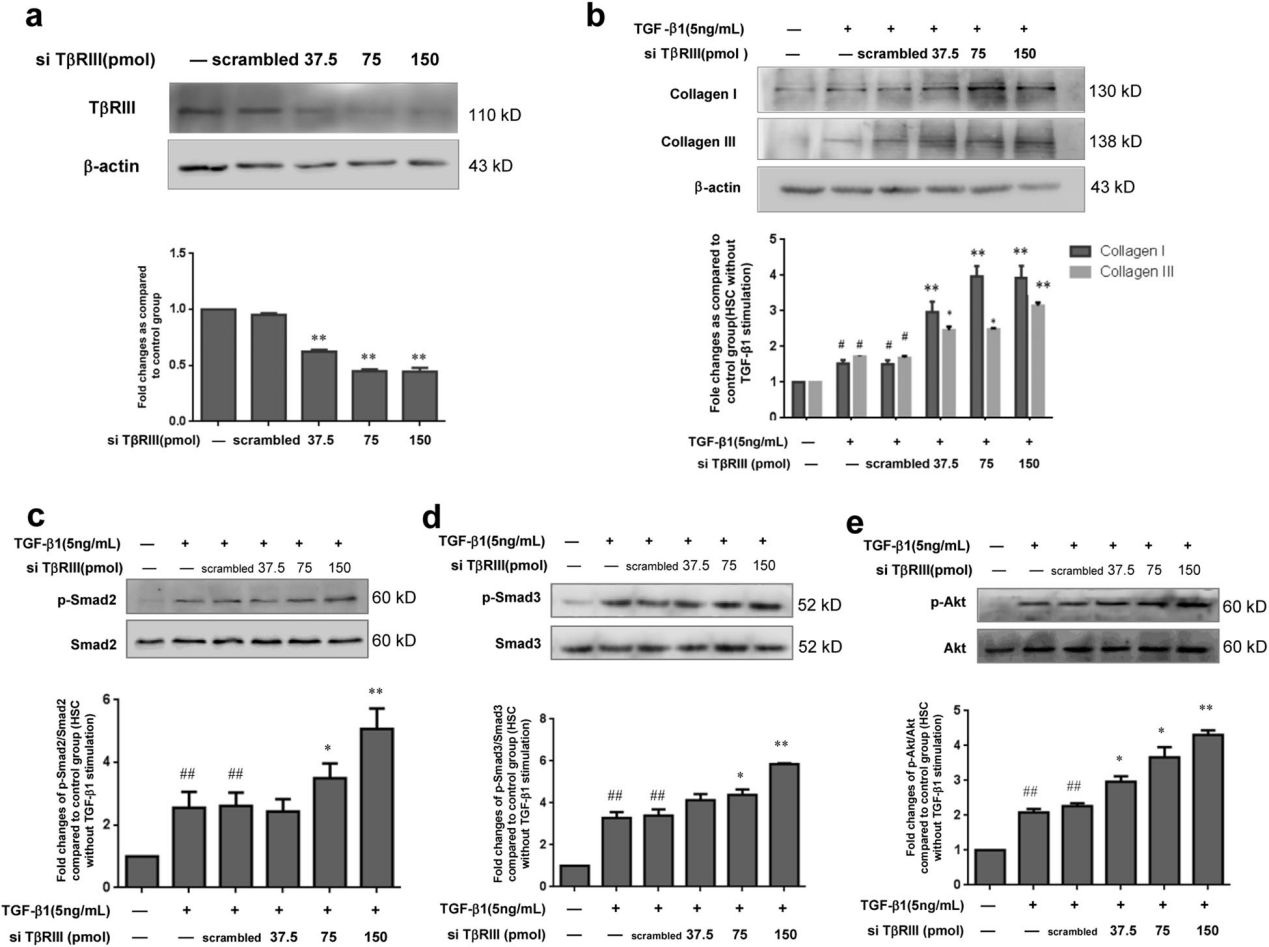
Gene silencing of TβRIII enhanced TGF-β1-induced collagen production. **a** Protein level of TβRIII in the stable HSC-T6 cells transfected with TβRIII siRNA. **b** The increased collagen I and collagen III levels were observed in TβRIII siRNA transfected HSCs. **c-e** Effects of transfecting TβRIII siRNA on activation of Smad2, Smad3 and Akt pathway in TGF-β1 stimulated HSCs. Down-regulation of TβRIII resulted in the increased activation of Smad2, Smad3 and Akt. The changes in p-Smad2, p-Smad3, p-Akt are expressed as ratio of phosphorylated/unphosphorylated forms and are shown as a bar diagram. ^#^*P* < 0.05, ^##^*P* < 0.01 vs. control group; *P < 0.05, **P < 0.01 vs. scrambled siRNA group.

To further investigate a role of TβRIII in β-arrestin2 deficiency-mediated collagen suppression, we next used siRNA targeting TβRIII to block the expression of TβRIII in HSCs that were stimulated with TGF-β1. Western blot results revealed that siRNA against TβRIII decreased TβRIII protein expression in HSC-T6 cells (Fig. 6a). Further experiments showed that when the expression of TβRIII was reduced by TβRIII siRNA, the collagen I and III levels in HSCs were increased compared with those in TGF-β1-treated cells that were not transfected (Fig. 6b). We also examined TGF-β1 downstream signalling proteins that participate in TβRIII-mediated HSC collagen production. Forty-eight hours after transfection with TβRIII siRNA, HSCs were stimulated with TGF-β1 for 0.25 or 4 h, and the levels of p-Smad2, p-Smad3 and p-Akt were increased in cells with low TβRIII expression compared with those of cells that were transfected with the scrambled siRNA (Fig. 6c–e). These results indicate that downregulation of TβRIII expression affects collagen production in HSCs through upregulation of TGF-β1 signalling.

### The β-arrestin2/TβRIII interaction regulates collagen production in vitro

**Fig. 7:**
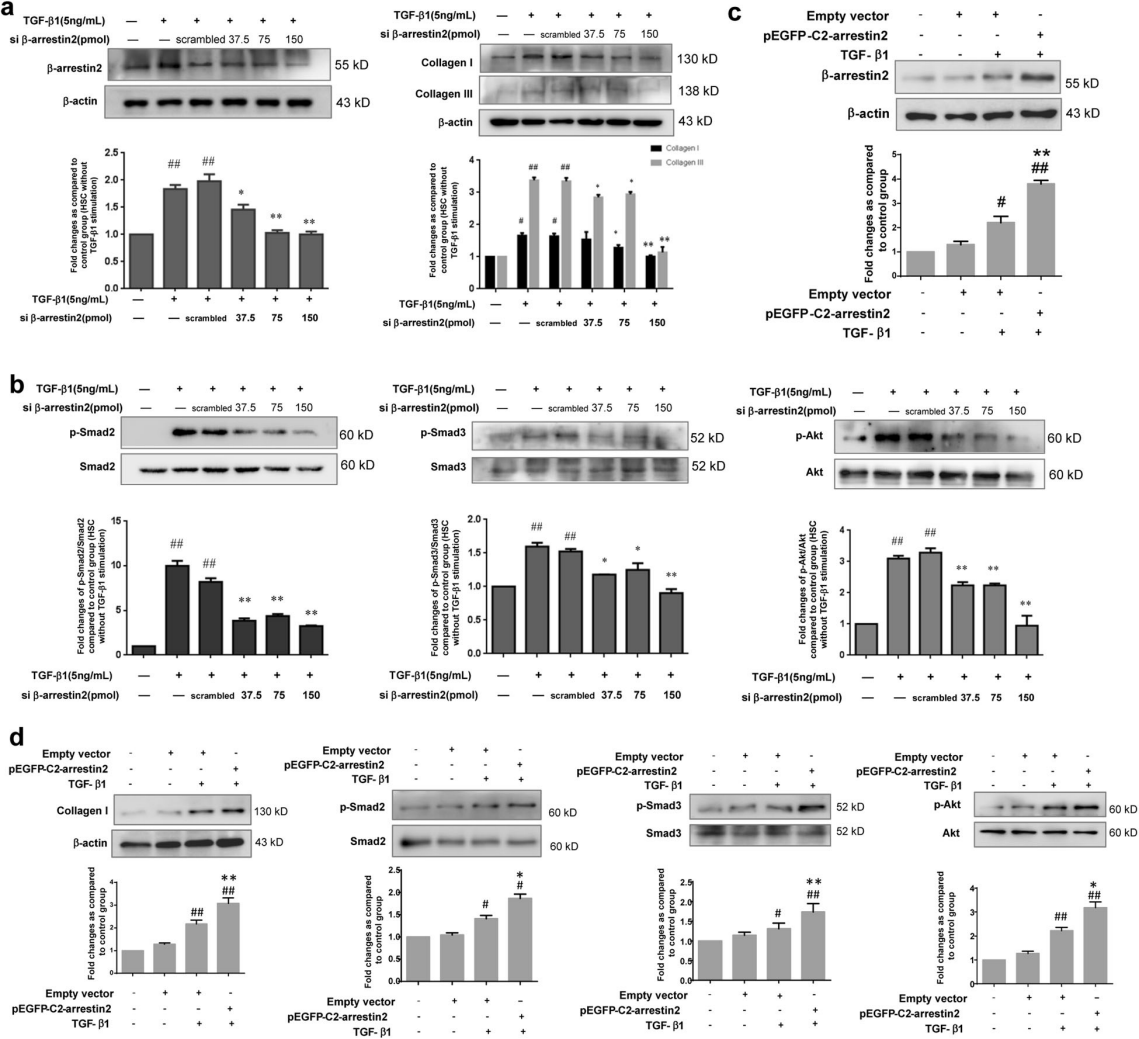
β-arrestin2 regulates collagen production via TGF-β1 downstream pathway. **a** Expression of β-arrestin2, collagen I and collagen III in TGF-β1 stimulated HSCs after transfecting β-arrestin2 siRNA, and the protein levels were normalized to β-actin for the Western blots from the same lysate. **b** Effects of transfecting β-arrestin2 siRNA on activation of Smad2, Smad3 and Akt pathways in TGF-β1 stimulated HSCs. ^#^*P* < 0.05, ^##^*P* < 0.01 vs. control group; *P < 0.05, **P < 0.01 vs. scrambled siRNA group. **c** β-arrestin2 was overexpressed in LX-2 cells, a human immortalized HSC line, by transfected the plasmid encoding pEGFP-C2-β-arrestin2. **d** Overexpression of β-arrestin2 significantly promoted collagen I production and activation of Smad2, Smad3, Akt in LX-2 cells. The changes in p-Smad2, p-Smad3, p-Akt are expressed as ratio of phosphorylated/unphosphorylated forms. ^#^*P* < 0.05, ^##^*P* < 0.01 vs. empty vector group; *P < 0.05, **P < 0.01 vs. TGF-β1 group.

Since TGF-β1 signalling was decreased during ECM production in β-arrestin2^−/−^ mice, the role of β-arrestin2 in this process remains poorly understood. Thus, we focused our study on the possible role of β-arrestin2 in HSC collagen production upon TGF-β1 stimulation in vitro. For this purpose, we first transfected β-arrestin2 siRNA into HSC-T6 cells to determine the effect of endogenous β-arrestin2 on collagen production. β-arrestin2 protein expression was significantly reduced, as determined by western blotting. Further experiments showed that when the expression of β-arrestin2 was reduced by β-arrestin2 siRNA, the collagen I and III levels in HSC-T6 cells were significantly decreased, which correlated with a decrease in the phosphorylation of Smad2, Smad3 and Akt (Fig. 7a, b). These findings indicate that the above changes in collagen production in TGF-β1-stimulated HSCs were inhibited when β-arrestin2 was knocked down. These in vitro data support the in vivo studies in which deficiency of β-arrestin2 correlated with reduced collagen production.

Because we observed that the loss of β-arrestin2 was closely associated with decreased collagen levels in HSCs, we hypothesized that overexpression of β-arrestin2 in HSCs promotes collagen production. Thus, we transfected a plasmid encoding pEGFP-C2-β-arrestin2 in LX-2 cells, a human immortalized HSC line. Successful overexpression of β-arrestin2 was confirmed by Western blotting (Fig. 7c). β-arrestin2 overexpression increased collagen I levels. Furthermore, the expression of phosphorylated Smad2, Smad3 and Akt was significantly increased when β-arrestin2 was overexpressed (Fig. 7d). Our results suggest that β-arrestin2 promotes HSC collagen production by positively regulating the TGF-β1 pathway.

**Fig. 8:**
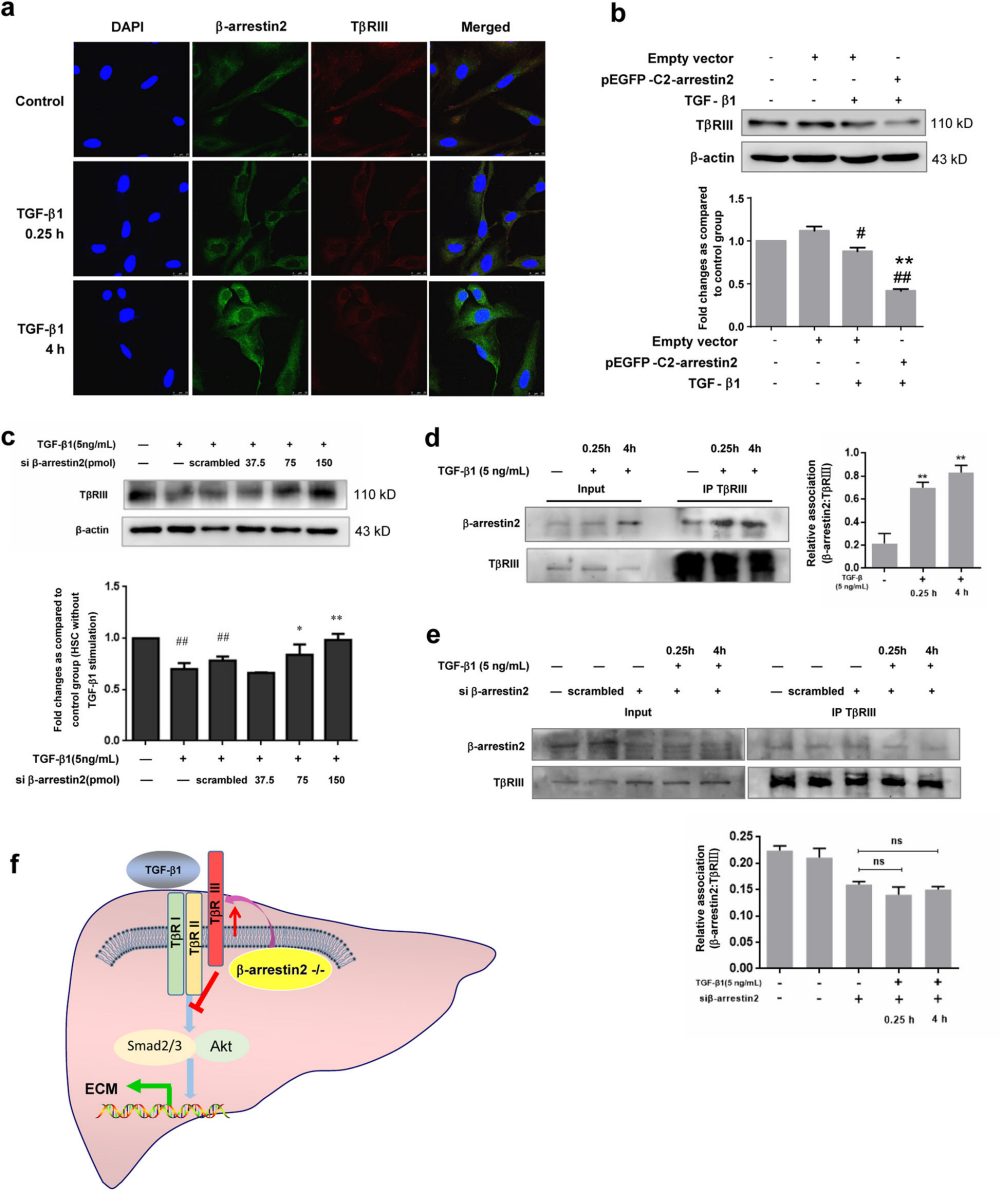
β-arrestin2 regulates collagen production via TβRIII. **a** The localization of β-arrestin2 and TβRIII in HSCs examined by immunofluorescent confocal microscopy. Cells were stained with Alex Fluor 488 donkey anti-mouse antibody (to detect β-arrestin2, in green), Alex Fluor 555 donkey anti-rabbit antibody (to detect TβRIII, in red), DAPI (to detect DNA, in blue). Scale bar = 25 μm. **b** Overexpression of β-arrestin2 significantly inhibited TβRIII expression in LX-2 cells upon TGF-β1 treatment. ^#^*P* < 0.05, ^##^*P* < 0.01 vs. empty vector group; **P < 0.01 vs. TGF-β1 group. **c** The increased expression of TβRIII in TGF-β1 stimulated HSCs was observed after transfecting β-arrestin2 siRNA. ^##^*P* < 0.01 vs. control group; *P < 0.05, **P < 0.01 vs. scrambled siRNA group. **d** Co-immunoprecipitation experiments of β-arrestin2 and TβRIII in HSCs. **P < 0.01 vs. without TGF-β1 treatment group. **e** After knockdown of β-arrestin2, the cell lysates were subjected to co-immunoprecipitation and western blot analysis. The ns indicates *P* > 0.05 vs. transfected with β-arrestin2 siRNA and without TGF-β1 treatment group. **f** Model depicting the role of β-arrestin2 in regulation of TGF-β1 signalling and collagen production.

Finally, we decided to study the putative role of the β-arrestin2/TβRIII interaction in TGF-β1-induced HSCs. The expression and subcellular localization of β-arrestin2 and TβRIII were confirmed by immunofluorescence confocal microscopy (Fig. 8a). Immunofluorescent analysis showed that β-arrestin2 protein was diffusely expressed predominantly in the cytoplasm of untreated HSCs and co-expressed with TβRIII. However, expression of β-arrestin2 was distributed in the cytoplasmic membrane of HSCs after TGF-β1 stimulation for up to 4 h. Moreover, the immunofluorescent staining intensity of β-arrestin2 was increased upon TGF-β1 treatment, which was in consistent with the Western blot analysis. These observations suggest that co-expression of β-arrestin2 and TβRIII resulted in their co-localization in the cytoplasm of HSCs. To investigate whether β-arrestin2 has a role in regulating TβRIII, we again used siRNA targeting β-arrestin2 or expression vectors carrying β-arrestin2 in HSCs. A decrease in TβRIII was observed in β-arrestin2-overexpressing cells compared with cells that were transfected with the empty vector. TβRIII expression was increased in cells that were treated with β-arrestin2 siRNA and TGF-β1 (Fig. 8b, c). We next examined whether β-arrestin2 interacts with TβRIII by co-immunoprecipitation. Initially, β-arrestin2 and TβRIII were co-expressed in HSCs, as determined by co-immunoprecipitation. Immunoprecipitation with TβRIII antibody resulted in the co-precipitation of β-arrestin2. Enhanced co-immunoprecipitation of β-arrestin2 and TβRIII was observed in HSCs upon TGF-β1 treatment (Fig. 8d), while the β-arrestin2/TβRIII interaction did not significantly change after siRNA-mediated silencing of β-arrestin2 in TGF-β1-stimulated HSCs (Fig. 8e). These results suggest that decreased β-arrestin2 expression in HSCs may be through increasing TβRIII expression and downregulating its interaction with TβRIII, thus inhibiting TGF-β1 signalling and collagen production (Fig. 8f).

## Discussion

Increasing evidence suggests that β-arrestins trigger signalling cascades independent of G-protein activation, and mediate many intracellular signalling networks, such as Notch, Wnt and TGF-β pathways, and downstream kinases including MAPK and PI3K^6,12^. These signalling pathways have been shown to be involved in the formation of fibrosis. Our previous studies found that with the exacerbation of liver fibrosis, expression of β-arrestin2 but not β-arrestin1 in liver tissues is increased^10^. However, its clinical relevance in regard to the progression of hepatic fibrosis and collagen production has never been clarified. Therefore, our present study was designed to investigate the role of β-arrestin2 deficiency in hepatic fibrosis. The results further demonstrate that β-arrestin2 plays a crucial role in collagen production and that β-arrestin2 deficiency ameliorates liver fibrosis.

Different models of hepatic fibrosis have been used to study the molecular pathogenesis of this disease. The PS-induced liver fibrosis model in rats manifests changes that are similar to those found in liver diseases in humans^13^. The current study demonstrated that both β-arrestin2, TGF-β1 and collagen were increased during fibrosis development in this model. Furthermore, β-arrestin2 expression in the fibrotic liver was positively associated with collagen I and collagen III levels. These results were further validated in the CCl_4_-induced chemical liver fibrosis model. Importantly, β-arrestin2 levels were also elevated in human fibrosis samples. The results demonstrate that induction of β-arrestin2 occurs in two different animal models of hepatic fibrosis and in human fibrosis samples and is associated with increased collagen levels.

Some studies have shown aberrant protein expression of β-arrestin2 associated with fibrosis-associated diseases^14^. For instance, Fan et al.^15^ demonstrated that in experimental ulcerative colitis (intestinal fibrosis) rats, the expression of β-arrestin2 was obviously decreased in the colonic mucosa compared with those of the normal control group. In contrast, an increasing number of studies have indicated that β-arrestin2 expression is increased in some fibrotic diseases. β-arrestin2^−/−^ mice are protected from excessive collagen deposition in a bleomycin-induced lung fibrosis model^8^. Moreover, increased expression of β-arrestin2 protein was observed in cystic fibrosis cells^16^. However, to date, the role of β-arrestin2 deficiency in liver fibrosis has not been investigated. Our current studies demonstrate that β-arrestin2^−/−^ mice showed minimal collagen deposition and less hydroxyproline in the liver than WT mice. β-arrestin2 deficiency also reduced serum transaminase activities, which indicates that β-arrestin2^−/−^ mice are protected from CCl_4_-induced liver injury. Since oxidative stress and subsequent lipid peroxidation participate in CCl_4_-induced hepatic fibrosis^17^, we examined oxidative stress parameters. Increased liver MDA levels and decreased SOD and GSH were detected in the WT model mice. β-arrestin2^−/−^ mice had significantly elevated GSH levels and decreased MDA levels, compared with those of WT mice. These data demonstrate that β-arrestin2 depletion inhibits lipid peroxidation and restores the antioxidative defence system in hepatic fibrosis.

Increasing evidence suggests that inappropriate inflammation drives the progression of fibrosis, and some studies have concentrated on the imbalance between Treg cells and other effector T cells as a reason for this inappropriate inflammation^18,19^. It has been reported that the Treg/Th17 balance might influence fibrosis progression in hepatitis B virus-related liver fibrosis via an increase in liver injury and promotion of HSC activation^20^. Intriguingly, β-arrestin2 also plays a role in inflammation and the immune response. β-arrestin2 induced the production of IL-17 and CD4^+^ T lymphocyte expression in a mouse asthma model^21^. In the OVA-induced murine model of allergic asthma, pulmonary eosinophil and CD4 T cell infiltration, as well as IL-4, IL-6, IL-13 and TNF-α levels, were all enhanced in WT but not in β-arrestin2^−/−^ mice^22^. To gain in vivo evidence of the effect of β-arrestin2 on T cell activation during liver fibrosis, we determined the frequencies of T cell subsets in the splenic lymphocytes of mice. The frequencies of activated T cells and the Th17/Treg ratio were significantly increased in WT mice after CCl_4_ treatment, while naïve T cells were decreased. β-arrestin2 deficiency contributed to the reduction in activated T cells and the Th17/Treg ratio, and the enhancement of naïve T cells in the spleens of fibrotic mice. These results indicate that the inflammatory response after CCl_4_ treatment in β-arrestin2^-/-^ mice was attenuated.

Our data suggest a previously unrecognized important role for β-arrestin2 deficiency in ameliorating liver fibrosis and regulating collagen formation *in vivo*. However, we cannot exclude other underlying mechanisms of β-arrestin2-mediated signalling pathways in the progression of hepatic fibrosis because of the complexity of β-arrestin2 signalling cascades. TGF-β1 is the most potent liver pro-fibrotic cytokine^23^. Although Smad-mediated signalling is well described as a significant mechanism of TGF-β1 signalling, additionally, Smad-independent pathways, such as MAPK, Akt, NF-κB pathways also participate in TGF-β1 signalling^3,24^. We examined whether β-arrestin2-regulated collagen formation was associated with TGF-β1 signalling. Our results indicate that both TGF-β1 and its downstream signalling molecules p-Smad2, p-Smad3, p-Akt were decreased in β-arrestin2^−/−^ mice compared with those of WT mice. In vitro, TGF-β1-stimulated HSCs were used to further explore the role of β-arrestin2 in collagen synthesis. As expected, the collagen levels and activation of Smad2, Smad3, Akt were increased upon TGF-β1 stimulation. Moreover, β-arrestin2 expression gradually increased. To further investigate a role of β-arrestin2 in collagen production, we utilized siRNA targeting β-arrestin2 or transfected plasmids encoding β-arrestin2 in HSCs. Results showed that the collagen level and p-Smad2, p-Smad3, p-Akt were significantly increased when β-arrestin2 was overexpressed, while collagen production and Smad2, Smad3, Akt activation induced by TGF-β1 were inhibited when β-arrestin2 was knocked down in HSCs. These data together suggest that β-arrestin2 promotes HSCs collagen production by positively regulating the TGF-β1 pathway in HSCs.

TGF-β regulates diverse cellular processes through a heteromeric complex of TβRI, TβRII and TβRIII. TGF-β ligands bind to constitutively active TβRII on the cell surface, activating TβRI and then forming heteromeric complexes to induce downstream signal^3^. TβRIII, which lacks intrinsic enzymatic activity, is the most abundantly expressed TGF-β superfamily receptor. TβRIII has primarily been considered to function as a coreceptor. However, recent studies have identified that TβRIII has a complex and context-dependent role in regulating TGF-β superfamily signalling and disease development. TβRIII functions as a potent inhibitor of TGF-β signalling by preventing type I-type II receptor complex formation^3^. In NIH/3T3 fibroblasts that stably expressed TβRIII, Smad2/3, Akt, ERK phosphorylation and procollagen type I expression were inhibited^25^. In several cancers, including breast cancer, prostate cancer, and lung cancer, TβRIII expression is reduced or even lost^26^. Our previous studies found that TβRIII expression was decreased in hepatocellular carcinoma (HCC) patient tissues, and knockdown of TβRIII promoted the migration and invasion of HCC cells^27^. Our present results show that TβRIII expression in PS-injected rats significantly decreased with the progression of fibrosis, which correlated with an increase in β-arrestin2 levels. In addition, TβRIII was downregulated in human fibrotic samples. Corroborating the results of the in vitro experiments, HSCs that were transfected with TβRIII siRNA showed high collagen I and collagen III expression and an increase in Smad2, Smad3 and Akt phosphorylation. These results indicate that downregulation of TβRIII expression affects collagen production in HSCs through upregulation of TGF-β1 signalling.

β-arrestin2 functions as a multiprotein scaffold to coordinate complex signal transduction networks. Recent studies have indicated that β-arrestin2 binds TβRIII and is involved in its clathrin-independent/lipid raft pathway-dependent internalization^28,29^. TβRIII, through its interaction with β-arrestin2, activates Cdc42 and inhibits epithelial and cancer cell migration^30^. TβRIII expression inhibits TGF-β-mediated Smad2/3 nuclear translocation and transcriptional activation in MDA-MB-231 cell lines^31^. How might β-arrestin2 deficiency negatively regulate TGF-β1 signalling through its interaction with TβRIII? Our current results showed that TβRIII expression was increased in β-arrestin2^−/−^ mice compared with that of WT mice with CCl_4_-induced liver fibrosis. In addition, a decrease of TβRIII in β-arrestin2-overexpressing HSCs was observed. Conversely, TβRIII expression was increased in HSCs that were treated with β-arrestin2 siRNA and TGF-β1. Both co-immunoprecipitation and fluorescence confocal studies demonstrated the interaction between β-arrestin2 and TβRIII in HSCs. Previous studies have proposed a model in which the binding of TβRII and TβRI to TβRIII competes with the construction of the TβRII/TβRI complex, thus suppressing signalling to the Smad pathway^31^. The findings of our studies raised the possibility that β-arrestin2 deficiency enhances TβRIII expression and suppresses TGF-β1 signalling, thereby reducing collagen production and ameliorating liver fibrosis.

In summary, we provide evidence that β-arrestin2 deficiency ameliorated liver fibrosis in mice. Interfering with the expression of β-arrestin2 in HSCs inhibited collagen deposition through negative regulation of the TGF-β1 downstream pathway. Taken together, these findings suggest that locally delivered β-arrestin2 inhibitors may be a potential strategy for treating liver fibrosis.

## Materials and methods

### Animals

All animal experiments were conducted according to the guidelines of the Animal Care and Use Committee of Anhui Medical University, and the experiments were authorized by the Ethics Review Committee for Animal Experimentation of the Institute of Clinical Pharmacology, Anhui Medical University. Male Wistar rats weighing 120 ± 10 g were obtained from the Shanghai BK Experimental Animal Centre (Grade II, Certificate No. D-65). β-arrestin2^−/−^ mice (C57BL/6 background) were purchased from Jackson Laboratory (Maine, USA). Male and female mice were evaluated, and the control mice were age- and sex-matched littermates. Each mouse was genotyped at 21 days after birth as previously described^32^. The animals were housed in a pathogen-free room with a constant temperature of 23 ± 3 °C, humidity of 50 ± 20%, and a 12 h/12 h light/dark cycle. All animals were allowed free access to standard chow and tap water ad libitum throughout the experiment.

### Animal models of liver fibrosis

Two animal experimental models of liver fibrosis were used for this study: PS administration and CCl_4_ administration. The rats were randomly allocated into the normal control group and PS model group. Rats in the PS model group were intraperitoneally injected with PS at a dose of 0.5 mL/rat twice a week for a total of 16 weeks^33^. Rats in the normal control group were injected with the same amount of saline solution. After 3, 6, 9, 12 and 16 weeks of injections, eight rats in the PS group were sacrificed under anaesthesia. The liver samples were collected for histopathological staining and Western blot analysis.

For CCl_4_ experiments, CCl_4_ (Shanghai Lingfeng Chemical Factory, Shanghai, China) was diluted in olive oil (Sigma, MO, USA) at a ratio of 1:9 and intraperitoneally injected (5 mL/kg body weight) into 6- to 8-week-old β-arrestin2^−/−^ and WT C57BL/6 mice (*n* = 8 per group). This administration was conducted twice a week for up to 6 weeks to establish liver fibrosis^34^. Age- and sex-matched control mice were treated twice weekly with similar volumes of olive oil injected i.p. The mice were sacrificed 6 weeks after initiation of the experiment.

### Human samples

Specimens from 28 cirrhosis patients with different degrees of fibrosis were extracted during surgeries and collected at the Affiliated Hospital of Anhui Medical University (Hefei, China). The control group comprised eight patients with intrahepatic biliary lithiasis. Liver histological examination revealed normal histology or minimal changes. All experimental procedures were approved by the research ethics committee of Anhui Medical University (No. 20131323). All patients participated after providing written informed consent. This study was conducted according to the guidelines formulated by the Science Council of China.

### Cell culture conditions and cell models

A rat HSC cell line (HSC-T6) or human immortalized HSC cell line (LX-2) was selected for in vitro studies, and the cell lines were acquired from the Cell Bank of the Chinese Academy of Sciences (Shanghai, China). The cells were cultured in DMEM (Life Technologies Inc., CA, USA) containing 10% foetal calf serum (HyClone, UT, USA) in a humidified atmosphere with 5% CO_2_ at 37 °C. The HSC cell lines possessed a fibroblast-like morphology and specific expression of α-smooth muscle actin^35^. Treatment of these HSCs with TGF-β1 triggered the morphological transition, which was concomitant with increased ECM synthesis^36^.

### Histology and immunohistochemical staining

HE staining of the liver tissues was conducted according to standard protocols. Immunohistochemical staining was performed as previously described^10^. Liver sections were dewaxed, rehydrated and subjected to antigen retrieval. Then, the sections were placed in 3% H_2_O_2_ in methanol for 10 min. After blocking, the sections were incubated with primary antibodies against β-arrestin2 and TβRIII (Santa Cruz Biotechnology, CA, USA) at specific dilutions for 1 h at 37 °C. Then, the immunoreactivity of the antibodies was detected with the streptavidin/peroxidase (SP) method (Zhongshan Goldenbridge Biotechnology Co., LTD, Beijing, China), and diaminobenzidine (DAB) was used to visualize the reaction. After counterstaining with haematoxylin solution, the sections were viewed under an Olympus BX53 microscope (Olympus Optical Co., Ltd., Tokyo, Japan). A negative control was performed using the same staining procedure but omitting the primary antibodies. Semiquantitative analysis was performed using Image-Pro Plus software (Media Cybernetics, USA). For each slide, five random fields were analysed.

### Analysis of serum transaminase activities

Serum samples were collected from all mice, and the transaminase activities of ALT and AST were evaluated by commercial kits (Jiancheng Biologic Co., Nanjing, China) according to the instructions.

### Determination of hydroxyproline levels in the liver

Approximately 100 mg of liver tissue was collected to determine the hydroxyproline level as described^37^. The level of hepatic hydroxyproline is an indirect indicator of tissue collagen levels and is presented as mg/g wet tissue.

### Analysis of antioxidase and lipid peroxidation

Hepatic tissues were rinsed with cold saline solution and subsequently homogenized on ice. After centrifugation at 4 °C and 1000×*g* for 15 min, the supernatants were collected. The activities of SOD and GSH were measured to evaluate the antioxidases, and the results are presented as the units of SOD per milligram of hepatic tissue or GSH μmol/g protein. The lipid peroxidation state of the liver was detected by determining the MDA level, which is presented as nmol/mg protein. The procedures were conducted according to the kit instructions (Jiancheng Biologic Co., Nanjing, China).

### Preparation of splenic lymphocytes and T cell subset analysis

After the mice were anaesthetized and sacrificed, single-cell spleen suspensions were harvested by mechanical separation of spleen tissue through nylon mesh. Lymphocytes were obtained from the gradient interphase. Then, the cells were rinsed with PBS three times and stained with specific fluorescent antibodies, including anti-CD4-FITC, anti-CD25-APC (eBioscience, CA, USA), anti-CD62L-PE, and anti-CD69-PE (Miltenyi Biotec, Bergisch Gladbach, Germany), in the dark at 4 °C for 20 min. For analysis of the Treg and Th17 subsets, the cells were fixed and permeabilized, followed by incubation with anti-Foxp3-PE and anti-IL-17-PE antibodies (eBioscience, CA, USA). Afterwards, the cells were washed and resuspended in PBS, and the prepared samples were analysed on a BD FACSVerse flow cytometer (BD Biosciences, NJ, USA).

### siRNA transfection and DNA transfection

For β-arrestin2 or TβRIII knockdown, HSC-T6 cells were seeded in 6-well plates and transfected with specific siRNA duplexes purchased from GenePharma Company (Shanghai, China) targeting β-arrestin2 and TβRIII RNA. A scrambled RNA duplex served as a negative control. The HSCs were incubated for 48 h after transfection and then harvested for Western blot analysis.

For overexpression of β-arrestin2, a pcDNA3 expression plasmid encoding pEGFP-C2-β-arrestin2 was used in this study, which was kindly provided by Dr. Yang K. Xiang of the University of California, Davis. LX-2 cells were grown in 6-well plates and transiently transfected with the β-arrestin2 overexpression vector using Lipofectamine 3000 (Invitrogen Life Technologies, CA, USA) according to the manufacturer’s protocols. Each well contained 5 μg of DNA.

### Western blotting analysis

Total protein was harvested from hepatic tissues or HSCs. Western blotting was conducted as previously described^38^. The primary antibodies for β-arrestin2 (sc-13140), TβRII (sc-17792), TβRIII (sc-28975), TGF-β1 (sc-52893), collagen III (sc-514601), β-actin (sc-69879) were purchased from Santa Cruz Biotechnology Inc (Santa Cruz, CA, USA), for collagen I (RT1152) from HuaAn Biotechnology Co., Ltd., (Hangzhou, China), for p-Smad2 (#3108), Smad2 (#5339), p-Smad3 (#9520), Smad3 (#9523), p-Akt (#4058), Akt (#4691) from Cell Signaling Technology (Danvers, MA, USA). Specific proteins were detected by chemiluminescence system.

### Immunofluorescence double-labelling assay

Cells were seeded in a six-well dish with poly-D-lysine-coated coverslips. After incubation overnight, the cells were starved and stimulated with TGF-β1 5 ng/mL (PeproTech, NJ, USA) for the indicated time. The cells were then fixed with 4% paraformaldehyde for 20 min, washed thrice with PBS and permeabilized with 0.1% Triton X-100 for 5 min. After that, the cells were incubated with 1% bovine serum albumin, followed by primary antibodies against β-arrestin2 and TβRIII overnight at 4 °C. The samples were subsequently incubated with a mixture of Alexa Fluor 555-conjugated anti-rabbit and Alexa Fluor 488-conjugated anti-mouse secondary antibodies (Life Technologies Inc., CA, USA) for 2 h in the dark. The samples were then mounted with a sealer containing DAPI, and the images were captured with a Leica SP8 laser scanning confocal microscope (Leica Biosystems, Wetzlar, Germany). β-arrestin2-positive expression is presented as green fluorescent foci, TβRIII-positive expression is presented as red fluorescent foci, and colocalization of these two proteins is presented as yellow fluorescent foci.

### Co-immunoprecipitation assay

Cells were collected in RIPA lysis buffer (Beyotime Biotechnology, Shanghai, China) supplemented with a mammalian protease inhibitor mixture (Biocolors, Shanghai, China). The cell lysate was immunoprecipitated (IP) with anti-TβRIII antibody, subsequently separated by SDS-PAGE and subjected to Western blotting analysis with anti-β-arrestin2 antibody (Santa Cruz Biotechnology, CA, USA). The assay was performed in accordance with standard procedures.

### Statistical analysis

Statistical analysis was carried out using SPSS software version 15.0 (SPSS Inc., Chicago, IL, USA). The data are collected from eight animals per group for in vivo studies and at least four independent experiments for in vitro studies, and are presented as the means and standard deviation of the mean unless otherwise indicated. Analysis of variance (ANOVA) and Student’s *t*-tests were used to identify significant differences between groups. The correlation between the expression of β-arrestin2 and collagen expression in liver tissues was performed by Pearson’s correlation analysis. Values of *P* < 0.05 were considered to be statistically significant.

## References

[1] Schuppan D, Ashfaq-Khan M, Yang AT, Kim YO (2018). Liver fibrosis: Direct antifibrotic agents and targeted therapies. Matrix Biol..

[2] Tsuchida T, Friedman SL (2017). Mechanisms of hepatic stellate cell activation. Nat. Rev. Gastroenterol. Hepatol..

[3] Zhang S, Sun WY, Wu JJ, Wei W (2014). TGF-beta signaling pathway as a pharmacological target in liver diseases. Pharmacol. Res..

[4] Vander AA, Cao J, Li X (2018). TGF-beta receptors: in and beyond TGF-beta signaling. Cell. Signal..

[5] Peterson YK, Luttrell LM (2017). The diverse roles of arrestin scaffolds in G protein-coupled receptor signaling. Pharmacol. Rev..

[6] Hu S (2013). Involvement of beta-arrestins in cancer progression. Mol. Biol. Rep..

[7] Nakaya M (2012). Induction of cardiac fibrosis by beta-blocker in G protein-independent and G protein-coupled receptor kinase 5/beta-arrestin2-dependent signaling pathways. J. Biol. Chem..

[8] Lovgren AK (2011). Beta-arrestin deficiency protects against pulmonary fibrosis in mice and prevents fibroblast invasion of extracellular matrix. Sci. Transl. Med..

[9] Xu H (2018). Beta-Arrestin-1 deficiency ameliorates renal interstitial fibrosis by blocking Wnt1/beta-catenin signaling in mice. J. Mol. Med. (Berl.)..

[10] Sun WY (2013). Depletion of beta-arrestin2 in hepatic stellate cells reduces cell proliferation via ERK pathway. J. Cell. Biochem..

[11] Shen H (2017). Thymic NF-kappaB-inducing kinase regulates CD4(+) T cell-elicited liver injury and fibrosis in mice. J. Hepatol..

[12] Luttrell LM (2013). Arrestin pathways as drug targets. Prog. Mol. Biol. Transl. Sci..

[13] Bai F (2018). Gypsophila elegans isoorientin-2”-O-alpha-l-arabinopyranosyl ameliorates porcine serum-induced immune liver fibrosis by inhibiting NF-kappaB signaling pathway and suppressing HSC activation. Int. Immunopharmacol..

[14] Gu YJ, Sun WY, Zhang S, Wu JJ, Wei W (2015). The emerging roles of beta-arrestins in fibrotic diseases. Acta Pharmacol. Sin..

[15] Fan H (2012). Role of beta2-adrenoceptor-beta-arrestin2-nuclear factor-kappaB signal transduction pathway and intervention effects of oxymatrine in ulcerative colitis. Chin. J. Integr. Med..

[16] Manson ME, Corey DA, Bederman I, Burgess JD, Kelley TJ (2012). Regulatory role of beta-arrestin-2 in cholesterol processing in cystic fibrosis epithelial cells. J. Lipid Res..

[17] Mortezaee K (2018). Nicotinamide adenine dinucleotide phosphate (NADPH) oxidase (NOX) and liver fibrosis: a review. Cell Biochem. Funct..

[18] Niu Y (2011). The balance between intrahepatic IL-17(+) T cells and Foxp3(+) regulatory T cells plays an important role in HBV-related end-stage liver disease. BMC Immunol..

[19] Zhu J, Paul WE (2010). Heterogeneity and plasticity of T helper cells. Cell Res..

[20] Cachem F (2017). The proportion of different interleukin-17-producing T-cell subsets is associated with liver fibrosis in chronic hepatitis C. Immunology.

[21] Liu Y (2011). Beta-arrestin2 stimulates interleukin-17 production and expression of CD4+ T lymphocytes in a murine asthma model. Iran. J. Allergy Asthma Immunol..

[22] Nichols HL (2012). Beta-Arrestin-2 mediates the proinflammatory effects of proteinase-activated receptor-2 in the airway. Proc. Natl Acad. Sci. USA.

[23] Walton KL, Johnson KE, Harrison CA (2017). Targeting TGF-beta mediated SMAD signaling for the prevention of fibrosis. Front. Pharmacol..

[24] Stewart AG, Thomas B, Koff J (2018). TGF-beta: Master regulator of inflammation and fibrosis. Respirology.

[25] Ahn JY, Park S, Yun YS, Song JY (2010). Inhibition of type III TGF-beta receptor aggravates lung fibrotic process. Biomed. Pharmacother..

[26] Turley RS (2007). The type III transforming growth factor-beta receptor as a novel tumor suppressor gene in prostate cancer. Cancer Res..

[27] Zhang S, Sun WY, Wu JJ, Gu YJ, Wei W (2016). Decreased expression of the type III TGF-beta receptor enhances metastasis and invasion in hepatocellullar carcinoma progression. Oncol. Rep..

[28] Chen W (2003). Beta-arrestin 2 mediates endocytosis of type III TGF-beta receptor and down-regulation of its signaling. Science.

[29] Finger EC, Lee NY, You HJ, Blobe GC (2008). Endocytosis of the type III transforming growth factor-beta (TGF-beta) receptor through the clathrin-independent/lipid raft pathway regulates TGF-beta signaling and receptor down-regulation. J. Biol. Chem..

[30] Mythreye K, Blobe GC (2009). The type III TGF-beta receptor regulates epithelial and cancer cell migration through beta-arrestin2-mediated activation of Cdc42. Proc. Natl Acad. Sci. USA.

[31] Tazat K, Hector-Greene M, Blobe GC, Henis YI (2015). TbetaRIII independently binds type I and type II TGF-beta receptors to inhibit TGF-beta signaling. Mol. Biol. Cell..

[32] Sun WY, Sun JC, Li XR, Peng WT, Wei W (2018). Breeding and genotype identification of Arrb2 gene knockout mice. Chin. Pharmacol. Bull..

[33] Sun WY, Wang L, Liu H, Li X, Wei W (2012). A standardized extract from Paeonia lactiflora and Astragalus membranaceus attenuates liver fibrosis induced by porcine serum in rats. Int. J. Mol. Med..

[34] Yang J (2017). MicroRNA-145 increases the apoptosis of activated hepatic stellate cells induced by TRAIL through NF-kappaB signaling pathway. Front. Pharmacol..

[35] Vogel S (2000). An immortalized rat liver stellate cell line (HSC-T6): a new cell model for the study of retinoid metabolism in vitro. J. Lipid Res..

[36] Martin-Mateos R (2019). Enhancer of zeste homologue 2 inhibition attenuates TGF-beta dependent hepatic stellate cell activation and liver fibrosis. Cell Mol. Gastroenterol. Hepatol..

[37] Sun WY, Wei W, Wu L, Gui SY, Wang H (2007). Effects and mechanisms of extract from Paeonia lactiflora and Astragalus membranaceus on liver fibrosis induced by carbon tetrachloride in rats. J. Ethnopharmacol..

[38] Gu YJ, Sun WY, Zhang S, Li XR, Wei W (2016). Targeted blockade of JAK/STAT3 signaling inhibits proliferation, migration and collagen production as well as inducing the apoptosis of hepatic stellate cells. Int. J. Mol. Med..

